# Ecological Niche Dimensionality and the Evolutionary Diversification of Stick Insects

**DOI:** 10.1371/journal.pone.0001907

**Published:** 2008-04-02

**Authors:** Patrik Nosil, Cristina P. Sandoval

**Affiliations:** 1 Zoology Department and Centre for Biodiversity Research, University of British Columbia, Vancouver, British Columbia, Canada; 2 Marine Science Institute, University of California Santa Barbara, Santa Barbara, California, United States of America; Lund University, Sweden

## Abstract

The degree of phenotypic divergence and reproductive isolation between taxon pairs can vary quantitatively, and often increases as evolutionary divergence proceeds through various stages, from polymorphism to population differentiation, ecotype and race formation, speciation, and post-speciational divergence. Although divergent natural selection promotes divergence, it does not always result in strong differentiation. For example, divergent selection can fail to complete speciation, and distinct species pairs sometimes collapse (‘speciation in reverse’). Widely-discussed explanations for this variability concern genetic architecture, and the geographic arrangement of populations. A less-explored possibility is that the degree of phenotypic and reproductive divergence between taxon pairs is positively related to the number of ecological niche dimensions (i.e., traits) subject to divergent selection. Some data supporting this idea stem from laboratory experimental evolution studies using *Drosophila*, but tests from nature are lacking. Here we report results from manipulative field experiments in natural populations of herbivorous *Timema* stick insects that are consistent with this ‘niche dimensionality’ hypothesis. In such insects, divergent selection between host plants might occur for cryptic colouration (camouflage to evade visual predation), physiology (to detoxify plant chemicals), or both of these niche dimensions. We show that divergent selection on the single niche dimension of cryptic colouration can result in ecotype formation and intermediate levels of phenotypic and reproductive divergence between populations feeding on different hosts. However, greater divergence between a species pair involved divergent selection on both niche dimensions. Although further replication of the trends reported here is required, the results suggest that dimensionality of selection may complement genetic and geographic explanations for the degree of diversification in nature.

## Introduction

The ecological niche is a key concept in ecology [1]–[10], and also plays a central role in evolutionary divergence. For example, during the process of ‘ecological speciation’, divergent selection between niches drives phenotypic divergence and the evolution of reproductive isolation [11]–[16]. Recent years have seen numerous examples of this process in a wide range of taxa [17]–[19]. Another increasingly realized factor is the often continuous nature of evolutionary divergence (even if the end point of the process is the development of a discontinuity) [20]–[39]. For example, phenotypic divergence can vary quantitatively [8], [12], [13], as can the magnitude of reproductive isolation [17]–[19], [33], [34], the degree of genotypic clustering [22], and the extent of lineage sorting in gene genealogies [23]–[28]. Different degrees of divergence can be thought of as arbitrary ‘stages’ of evolutionary divergence [29]–[31]. For example, divergence may proceed through stages such as polymorphism, population differentiation, ecotype and race formation, speciation, and post-speciational divergence [20]–[39]. We stress that arguments for the existence of stages of divergence do not rely on strict gradualism; shifts between stages could arise after long periods of little or no change, such that divergence is not always ongoing. Rather, the key point is that different taxon pairs may, at any point in time, exhibit different degrees of phenotypic, reproductive, and genetic divergence.

When it comes to the degree of divergence observed between taxon pairs, ecological differences between populations often result in some population differentiation, but in patterns inconsistent with strong evolutionary divergence, such as imperfect reproductive isolation, ongoing gene flow, and weak genotypic clustering [17]–[19], [29], [32]–[34]. Moreover, the collapse of distinct species pairs formed by selection has been documented [35]–[40], and some species pairs fail to diversify further following speciation [40]–[45]. What factors explain the extent to which divergent selection drives evolutionary divergence? Some well-considered factors are genetic architecture, time since divergence, and levels of gene flow [10], [17], [21], [46]–[48]. For example, the evolution of reproductive isolation during speciation is promoted by pleiotropic effects on reproductive isolation of genes under selection [47]–[52], physical linkage of genes under selection and those conferring reproductive isolation (perhaps facilitated by chromosomal inversions) [53]–[55], one-allele assortative mating mechanisms [18], [51], [52], [56], [57], increased time since divergence [17], [19], and geographic barriers to gene flow [17], [21], [33].

A less-considered explanation for variability in the degree of evolutionary divergence concerns the nature of the ecological niche, and more specifically, the number of niche dimensions (i.e., traits) subject to divergent selection [21], [50], [58]. Divergence between taxon pairs in a greater number of niche dimensions might promote phenotypic divergence and reproductive isolation, by causing population pairs to become more genetically divergent and to differ in a greater number of adaptive phenotypic traits. This, in turn, decreases the ecological fitness of hybrids and increases the probability that divergence occurs in genes that affect other forms of reproductive isolation (e.g., habitat and mate preference, genetic incompatibilities in hybrids). Thus, the ‘niche dimensionality’ hypothesis predicts a positive association between the number of niche dimensions subject to divergent selection and the degree of phenotypic, reproductive, and evolutionary divergence. We stress, however, that this hypothesis is not mutually exclusive from genetic, geographic, and time-based explanations for the degree of divergence, and these factors may interact to affect diversification (see discussion for consideration of causality).

**Figure 1:**
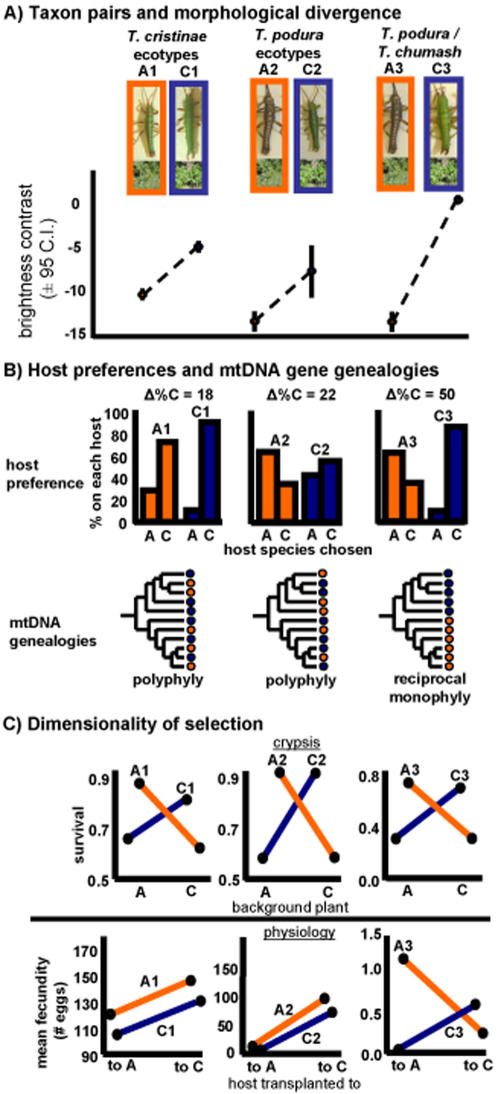
The number of niche dimensions subject to divergent selection and speciation of *Timema* walking-stick insects. Depicted are the two ecotype pairs and the species pair studied for the degree of phenotypic and evolutionary divergence in relation to the number of niche dimensions subject to divergent selection. A1 and C1 refer to ecotypes of *T. cristinae* (A = *Adenostoma* and C = *Ceanothus* hereafter). A2 and C2 refer to ecotypes of *T. podura*. A3 and C3 refer to the species pair *T. podura* and *T. chumash*, respectively. The ecotype pairs exhibit weaker divergence in morphology, host preference, and mtDNA than the species pair, and are also subject to divergent selection on fewer niche dimensions. A) Photographs of the three taxon pairs, and divergence in colour-pattern between them (brightness contrast, mean±95% C.I.). Host plants are also shown. B) Summary of divergence in host plant preferences and mtDNA (colours represent host plant use). Δ%C refers to the difference between each taxon pair in the percent of individuals choosing *Ceanothus* over *Adenostoma* in host preference trials [data from 78, 72, and the current study for *T. cristinae* ecotypes, *T. podura* ecotypes, and the species pair, respectively]. The phylogenetic trees are schematic for simplicity. The patterns depicted were robust to alternative methods for tree construction [75], [81 for details]. C) The nature of selection on crypsis and physiology for each taxon pair. For crypsis, the term ‘survival’ is used as a general y-axis label, representing the fitness of each insect host form on each host species. For ecotypes, the y-axes specifically represent 1- the proportion of insects eaten in predation trials with scrub jays [data from 72]. For the species pair, the y-axis specifically represents the proportion of each insect species on each host plant at the end of the field experiment (shown in more detail in Fig. 2). Further evidence that selection is exerted by visual predation stems from the observation that: (a) survival was measured using predation trials, or (b) divergent selection in manipulative field experiments was detected in the presence, but not in the absence, of visual predation [see also 70], [71]. For physiology, the y-axis represents lifetime fecundity in all cases (data on survival for the species pair are also reported in Fig. 2). The data depicted can be used to infer the presence versus absence of divergent selection, but should not be used to quantitatively compare the strength of selection (because somewhat different experimental procedures were used among taxa). For simplicity, error bars were removed for the current figure, but are depicted in Fig. 2. See text for statistical details.

The niche dimensionality hypothesis is not new, and has been discussed by various workers in the past [49], [50], [58]–[65]. However, it has received almost no focused empirical attention, despite its potential for complementing more geographic and genetic hypotheses. The idea itself stems largely from experimental evolution work using *Drosophila*, where a review by Rice and Hostert [50] noted that studies employing ‘multifarious’ divergent selection on multiple traits were more likely to result in the evolution of strong reproductive isolation than studies employing selection on a single trait. However, even in this work, to our knowledge no single study (i.e., one using the same species and experimental design) applied treatments that selected divergently on multiple versus single traits [50], [58]. Tests from nature are also lacking, owing in part to the difficulty of providing the required estimates of divergent selection for multiple taxon pairs at different stages of evolutionary divergence. Moreover, beyond just estimating such selection, a difficult task in its own right, testing the dimensionality hypothesis requires experimentally manipulating different sources of selection to isolate which niche dimensions are under divergent selection. Here we use such manipulative experiments, conducted in the wild, to test for patterns consistent with the niche dimensionality hypothesis in taxon pairs of herbivorous *Timema* walking-stick insects. Specifically, we consider ecotypes of *T. cristinae*, ecotypes of *T. podura*, and the species pair *T. podura/T. chumash* (Fig. 1). All these taxon pairs co-occur in sympatry or parapatry in some portions of their range, but are allopatric (i.e., separated by regions without suitable host plants) in others [33].

*Timema* are wingless insects that feed and mate on a variety of host-plant species in southwestern North America [66], [67]. Nymphs and adults rest on the leaves of their host during the day, and feed on the leaves at night. While resting on the plants, *Timema* are vulnerable to predation by birds and lizards [68]–[72]. The taxon pairs considered here use two distinct host-plant genera (*Ceanothus* spp: Rhamnaceae and *Adenostoma fasciculatum*: Rosaceae). These plants differ phenotypically: *Ceanothus* is relatively large, tree-like, and broad-leaved, while *Adenostoma* is small, bush-like, and exhibits thin, needle-like leaves. The host plants also belong to different families with differing phytochemistry [72]. Thus, two niche dimensions upon which divergent selection between hosts might act are cryptic morphology (to evade visual predators) and physiology (to adapt to plant chemistry). These are likely common axes of divergent selection in phytophagous insects [63], [73], [74].

Past experimental work focused exclusively on host-plant ecotypes of *T. cristinae* and of *T. podura*, defined by the host species they are found upon (*T. cristinae* regularly uses both host species, *T. podura* predominantly uses *Adenostoma*, but rare populations on *Ceanothus* exist) [33], [72]. Thus, different ecotypes feed on different host plant species, while multiple populations feeding on the same host species (in different geographic localities) comprise a single ecotype. In both species, different ecotypes exhibit moderate levels of evolutionary divergence. For example, they exhibit some differentiation in a whole suite of phenotypic traits, including colour, colour-pattern, body size, body shape, resting behavior, and pheromones [33], [68]–[72]. The ecotypes also exhibit partial, but incomplete, progress towards ecological speciation [33], [72], [75]–[80]. Specifically, multiple forms of reproductive isolation, such as habitat and sexual isolation, are stronger between pairs of populations using different hosts than between pairs of populations using the same host [33], [75], a signature of the process of ecological speciation [15]–[19]. Importantly, phenotypic divergence and reproductive isolation between the ecotypes has a genetic basis [33], [76]–[79].

However, a critical point is that although the ecotypes have diverged to some extent, speciation was not completed, and levels of divergence may not progress any further (Fig. 1). Experimental, morphological, and molecular data each indicate incomplete reproductive isolation between ecotypes, only weak genotypic clustering, and substantial gene flow between them, all indicative of incomplete speciation [33], [72], [75]–[80]. For example, divergence in host-plant preference, a common form of premating reproductive isolation between insect populations (i.e., ‘habitat isolation’) [15]–[18], is weak between ecotypes of both species. In fact, both ecotypes of both insect species generally prefer *Ceanothus* in preference trials, but with the *Ceanothus* ecotype exhibiting a slightly stronger preference for *Ceanothus* [72], [78], [79]. Ecotypes also exhibit weak phylogenetic divergence; they are not monophyletic in gene genealogies based upon mitochondrial (COI) DNA sequences, nuclear (ITS-2) DNA sequences, or AFLPs [75], [80], [81]. Finally, the ecotypes are considered conspecific in traditional taxonomic classification [66].

Experiments with the ecotypes have shown that divergent selection occurs for crypsis, but not for physiology [68]–[72]. Specifically, in the face of predation there are strong survival trade-offs between hosts such that each ecotype has much higher survival on its native host. In contrast, reciprocal transplant experiments in the absence of predation show that fecundity is higher on *Ceanothus* for both ecotypes of both species (i.e., no physiological trade-offs in host use). Thus, ecotypes of both species are subject to divergent selection only along the single niche dimension of crypsis.

We stress that these ecotypes are not necessarily in the act of differentiating further, and our test of the niche dimensionality hypothesis does not require that they will one day diverge to become distinct species (and thus we do not argue for such a scenario here). The key point is that the ecotypes exhibit moderate phenotypic and reproductive divergence, represent some intermediate stage of evolutionary divergence (i.e., prior to the completion of speciation), and are subject to divergent selection only on the axis of cryptic colouration. We also note that the ‘ecotype’ designation is somewhat arbitrary, other workers might consider them ‘morphs’. We retain the term ecotype, because the populations on different host plants differ in a whole suite of phenotypic traits [33], [68]–[72], different traits are sometimes independently inherited by different genes [33], [79], and the ecotypes also exhibit partial reproductive isolation [33], [72], [75]–[80]. Here we ask if a greater level of phenotypic and evolutionary divergence than observed between ecotypes is associated with selection on more niche dimensions (i.e., colour and physiology). Our interpretation of the results relies on the ecotypes representing a weaker degree of phenotypic and evolutionary divergence than the species pair discussed below. In contrast, our interpretation does not depend on whether the ecotypes are in the act of differentiating further, or if they are being maintained at their current level of differentiation as ‘polymorphism’ within species.

## Results

Here we report novel experimental data on divergent selection and phenotypic divergence between the species pair *T. chumash* and *T. podura*. The new data we report here are from *T. chumash* on *Ceanothus* (this species is not found on *Adenostoma* in nature) and *T. podura* on *Adenostoma* (*T. podura* is sometimes found on *Ceanothus* as well, but such populations are not treated in the between-species comparisons reported here). Both these species are monomorphic for colour within a particular host plant. In all the data presented here, *T. podura* were from *Adenostoma* and thus always a ‘brown’ morph, and *T. chumash* were from *Ceanothus* and thus always a ‘green’ morph.

Past molecular data suggest that these two species are more divergent from one another than the ecotypes discussed above, indicating that arguments for stronger divergence of the species pair do not rest solely upon traditional taxonomic classification. For example, sampling across both allopatric and sympatric sites shows that, unlike the ecotypes, the two species are reciprocally monophyletic for COI [81]. *T. podura* and *T. chumash* are also considered separate species under taxonomic classification, and are closely-related but unlikely to be sister-species (see discussion for the implications) [66], [67], [81].

**Figure 2:**
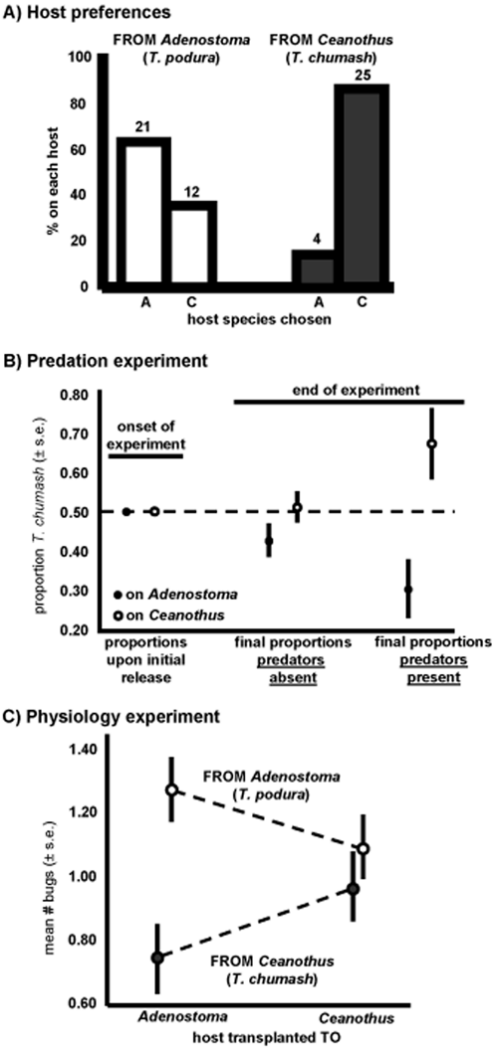
Host preferences and tests for divergent selection on crypsis and physiology. A) Host-plant preferences of *Timema* collected from *Adenostoma* (*T. podura*) or *Ceanothus* (*T. chumash*). Shown for each insect species is the percent of individuals choosing each host species in host choice trials. Numbers of individuals are denoted above the bars. Each *Timema* species preferred its native host. B) Results of the predation experiment. *T. podura* and *T. chumash* were released at equal proportion onto *Ceanothus* and *Adenostoma* bushes. Four weeks later the relative proportion of each insect species had diverged, but to a much larger extent when predation was present versus absent. Shown is the proportion of *T. chumash* (±1 S.E.) on each host species. C) Results of the physiology experiment. Norm-of-reaction plots showing means and standard errors (±1 S.E.) of survival of walking-sticks from *Ceanothus* or *Adenostoma* raised on their native or the alternative host-plant species. Survival was estimated as the mean number of insects observed alive within an enclosure, averaged across the multiple census periods. Physiological trade-offs in host plant use were evident.

Here we provide new morphological and experimental data on the distinctiveness of this species pair. The difference between the brightness of the exterior and central part of a *Timema's* body (‘brightness contrast’) is under strong divergent selection between hosts (*Ceanothus* versus *Adenostoma*) [71], and the degree of divergence in this trait thus represents one measure of the degree of divergent host-plant adaptation. The magnitude of divergence in brightness contrast is greater between *T. podura*/*T. chumash* than between ecotypes within species (Fig. 1; congruent patterns occur for most other morphological traits, Table 1). Trends in the same direction were detected for behavioral divergence in host-plant preference, where host preference experiments show that for the species pair, each insect species prefers its native host (insect species x host picked, **χ** ^2^ = 15.94, p<0.001, n = 62; *T. podura* and *T. chumash* picking *Adenostoma* in 64% and 14% of trials, respectively), a substantially greater degree of preference divergence than observed between ecotypes (Fig. 2). Thus, the species pair is more genetically, morphologically, and behaviorally distinct than the two ecotype pairs, and represents a greater overall level of evolutionary divergence.

**Table 1:** Morphological divergence between walking-stick taxon pairs.

	Taxon Pair																	
	*T. cristinae* ecotypes						*T. podura* ecotypes						*T. podura/T. chumash* species pair					
Trait	C mean	A mean	D	% Δ	F_1,683_	p	C mean	A mean	D	% Δ	F_1,49_	p	C mean	A mean	D	% Δ	F_1,172_	p
1	70.25	71.96	−1.71	**2.4**	2.50	0.11	47.02	35.11	11.91	**25.3**	3.27	0.08	77.89	35.11	42.78	**54.9**	200.92	0.000
2	76.24	68.67	7.57	**9.9**	40.72	0.000	51.36	26.41	24.95	**48.6**	13.14	0.001	55.15	26.40	28.75	**52.1**	49.95	0.000
3	43.49	39.44	4.05	**9.3**	78.06	0.000	40.33	25.22	15.11	**37.5**	18.71	0.000	54.16	25.22	28.94	**53.4**	181.73	0.000
4	67.46	66.97	0.49	**0.7**	0.35	0.55	52.05	44.50	7.55	**14.5**	1.71	0.20	71.64	44.50	27.14	**37.9**	108.67	0.000
5	74.29	61.97	12.32	**16.6**	106.12	0.000	59.05	38.67	20.38	**34.5**	13.71	0.001	61.79	38.67	23.12	**37.4**	31.18	0.000
6	49.02	50.89	−1.87	**3.7**	19.09	0.000	48.84	39.44	9.40	**19.2**	15.37	0.000	54.32	39.44	14.88	**27.4**	75.58	0.000
7	0.180	0.172	0.008	**4.4**	21.70	0.000	0.163	0.163	0.000	**0.0**	0.00	0.99	0.209	0.163	0.046	**22.0**	21.94	0.000
8	0.237	0.215	0.022	**9.3**	55.16	0.000	0.206	0.285	−0.080	**27.7**	58.03	0.000	0.276	0.285	−0.009	**3.2**	0.214	0.644
9	0.305	0.274	0.031	**10.2**	40.32	0.000	0.284	0.437	−0.15	**35.0**	84.95	0.000	0.350	0.437	−0.087	**19.9**	11.17	0.001
10	1.52	1.41	0.11	**7.2**	27.03	0.000	1.38	2.20	−0.82	**37.3**	114.07	0.000	1.57	2.20	−0.630	**28.6**	35.25	0.000
11	0.08	−0.40	0.49	**-**	55.15	0.000	−1.04	−0.36	−0.68	**-**	4.89	0.03	0.91	−0.36	1.27	**-**	17.87	0.000
12	0.40	0.14	0.26	**-**	18.14	0.000	−0.91	−3.04	2.13	**-**	22.73	0.000	−0.77	−3.04	2.28	**-**	62.67	0.000
13	0.16	−0.14	0.30	**-**	20.10	0.000	−1.48	−3.17	1.69	**-**	10.17	0.003	0.48	−3.17	3.65	**-**	429.08	0.000
14	0.50	−0.01	0.51	**-**	65.25	0.000	−0.08	−0.21	0.13	**-**	0.23	0.63	−1.04	−0.21	−0.83	**-**	7.11	0.008

We consider here ten traits that were examined in [71], as well as principle components (PC) axes generated from all these ten traits or from the colour variables only. Divergence in trait means between hosts was often statistically significant for all three taxon pairs (testing using F-ratios in ANOVA analyses), but the magnitude of divergence tended to be greater for the species pair than the ecotype pairs (particularly for colour traits, which are known to be under host-specific selection). Mean trait values are shown for *Ceanothus* (C) and *Adenostoma* (A), along with the difference between means (D = mean on *Ceanothus* minus mean on *Adenostoma*). Also shown is the percent difference between means (% **Δ**), calculated as 1–(smaller value/larger value). Thus, larger values of % **Δ** represent larger differences between taxon pairs (due to negative means, this calculation was not conducted for PC axes). This calculation is in bold to emphasize standardized differences between taxon pairs. Traits are as follows: 1 = body hue, 2 = body saturation, 3 = body brightness, 4 = stripe hue, 5 = stripe saturation, 6 = stripe brightness, 7 = head width, 8 = femur length, 9 = thorax width, 10 = body length, 11 = PC1 using all ten traits, 12 = PC2 using all ten traits, 13 = PC1 using only colour variables, 14 = PC2 using only colour variables.

There is no previous data on divergent selection in the *T. podura*/*T. chumash* species pair, prompting us to test for such selection at a site where the two species co-occur in sympatry. First, we tested for divergent selection on crypsis using a manipulative field experiment. An equal number of individuals from each insect species were released onto individual plants previously cleared of all *Timema* (40 plant individuals). This procedure was conducted for each host species, in both the presence and absence of predation (insects could disperse from all four of these treatments, see Methods). Changes through time in the proportion of insects that were *T. chumash* were then assessed (i.e., at the onset of the experiment this proportion was 0.5 for all plant individuals). In both the presence and the absence of predation, the proportion of *T. chumash* went down on its non-native host, and, conversely, went up on its native host (Fig. 2). However, the difference between the two host species in the final proportion of *T. chumash* was much greater in the presence of predation than in its absence (host x predation treatment interaction, F_1,40_ = 4.57, p = 0.039, ANOVA). When each predation treatment was considered separately, differences between hosts in the final proportion of *T. chumash* were highly significant in the presence of predation (main effects of host, F_1,20_ = 9.63, p = 0.006), but not in its absence (main effects of host, F_1,20_ = 2.02, p = 0.17). The small changes in the relative proportion of each species in the absence of predation may represent species-specific dispersal or physiological trade-offs in host-plant use, and the much larger effect in the presence of predation indicates that divergent selection on crypsis occurred [70], [71].

Second, we tested for divergent selection on physiology in the species pair *T. chumash*/*T. podura*. This involved raising nymphs of each species inside of mesh enclosures (which exclude visual predators), on each host in their natural habitat, in a field reciprocal-transplant experiment similar to the ones used to show a lack of physiological trade-offs between the ecotypes discussed above. The mean number of individuals surviving during the experiment was dependent upon an interaction between the species of *Timema* tested and the species of host plant that insects were transplanted to (Fig. 2). This pattern is consistent with divergent selection and fitness trade-offs [16], [62]. This interaction was significant in the best model chosen using AIC (F_1,87_ = 4.27, p = 0.042; Tables 2 and 3 for full results), and was significant or marginally insignificant in other models (Table 2). Specifically, on *Ceanothus* the mean number of individuals surviving was similar for the two *Timema* species (F_1,29_ = 0.78, p = 0.38). On *Adenostoma*, the number of individuals surviving was much greater for *T. podura* (whose native host is *Adenostoma*) than for *T. chumash* (who does not utilize *Adenostoma* in the wild) (F_1,29_ = 10.97, p = 0.002). Similar and even stronger trends were observed for fecundity, where interactions between *Timema* species and host-species transplanted to were statistically significant, and each *Timema* species exhibited higher fecundity on its native host than on its alternative host (Fig. 1; F_1,120_ = 11.14, p = 0.001 for interaction term in best fit AIC model; p<0.01 for interaction terms in second and third best AIC models, and in a full factorial model, Table 4). As for survival, fecundity differences between insect species were significant on *Adenostom*a (F_1,29_ = 8.50, p = 0.007), but not on *Ceanothus* (F_1,29_ = 3.35, p = 0.08). We note that significance testing aside, the best AIC model (off all possible models) for both fecundity and for survival included a *Timema* species by host species interaction term (Table 2). Thus, physiological trade-offs in host plant use occurred in the species pair, a clear contrast with the results from the ecotypes.

**Table 2:** AIC model selection results, with the different models sorted from best to worse fit.

Block	Species	Host	Block*Species	Block*Host	Species*Host	Block*Species*Host	AIC
Survival							
**X**	**X**	**X**			**X**		**213.2**
X	X						213.4
X	X	X		X	X		214.1
X	X	X	X		X		215.1
X	X	X					215.3
X	X		X				215.3
X	X	X	X	X	X		216.1
X	X	X		X			216.3
X	X	X	X				217.2
X	X	X	X	X	X	X	217.5
	X						217.9
	X	X			X		218.0
X	X	X	X	X			218.2
	X	X					219.9
X							221.2
X		X					223.2
X		X		X			224.2
		X					227.2
Fecundity							
	**X**	**X**			**X**		**396.3**
X	X	X			X		396.8
X	X	X	X		X		397.9
X	X	X		X	X		398.3
X	X	X	X	X	X		399.4
X	X	X	X	X	X	X	400.8
	X						403.9
X	X						404.5
	X	X					405.3
X							405.3
X	X		X				405.7
X	X	X					405.9
		X					406
X		X					406.7
X	X	X	X				407.1
X	X	X		X			407.5
X		X		X			408.2
*X*	*X*	*X*	*X*	*X*			408.7

The term of interest in testing for divergent selection is the Species^*^Host interaction. For survival, the interaction between Species and Host was significant in the best fit AIC model (F_1,87_ = 4.27, p = 0.042), and significant or marginally insignificant in other models (full factorial model, F_1,29_ = 3.03, p = 0.09; second and third best models picked by AIC that included the interaction, F_1,58_ = 3.72, p = 0.06, F_1,58_ = 4.04, p = 0.049, respectively). For fecundity, the interaction was significant in the best AIC model (F_1,120_ = 11.14, p = 0.001), and in other models (p<0.01 for interaction terms in second and third best AIC models, and in a full factorial model).

**Table 3:** Significance testing of the terms in the best AIC model (Table 2 for details), for survival and fecundity (the term of interest in testing for divergent selection is the Species*Host interaction, which was significant for both survival and fecundity, and is indicated in bold).

Source		Type III Sum of Squares	df	Mean Square	F	Sig.
Survival						
Intercept	Hypothesis	110.21	1	110.21	241.02	0.000
	Error	13.26	29	0.46		
Species	Hypothesis	3.33	1	3.33	10.94	0.001
	Error	26.50	87	0.31		
Host	Hypothesis	0.02	1	0.02	0.06	0.805
	Error	26.50	87	0.31		
Species * Host	**Hypothesis**	**1.30**	**1**	**1.30**	**4.27**	**0.042**
	Error	26.50	87	0.31		
Block	Hypothesis	13.26	29	0.46	1.50	0.077
	Error	26.50	87	0.31		
Fecundity						
Intercept		31.01	1	31.01	20.47	0.000
Species		4.41	1	4.41	2.91	0.091
Host		1.01	1	1.01	0.67	0.416
Species * Host		**16.88**	**1**	**16.88**	**11.14**	**0.001**
Error		175.70	116			
Total		229.00	120			
Corrected Total		*197.99*	*119*			

**Table 4:** Mean and standard error of fecundity of each *Timema* species when transplanted to *Ceanothus* versus *Adenostoma*.

Host transplanted to	*Timema* species	mean (s.e.)
*Ceanothus*	*T. chumash*	0.60 (0.21)
*Ceanothus*	*T. podura*	0.23 (0.10)
*Adenostoma*	*T. chumash*	0.03 (0.03)
*Adenostoma*	*T. podura*	1.17 (0.38)

Each insect species had higher fecundity on its native host, but differences between *Timema* species were significant only for *Adenostoma* (p = 0.08 on *Ceanothus* and p<0.01 on *Adenostoma*).

## Discussion

**Figure 3:**
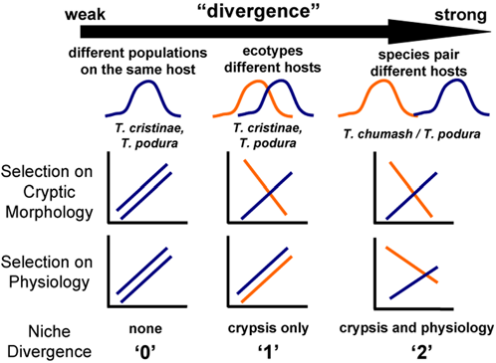
Summary of dimensionality of niche divergence in cryptic morphology and physiology, in relation to the diversification of *Timema*. The graphs depict fitness functions (y-axis is fitness, x-axis is trait value/habitat of origin), with crossing lines indicative of divergent selection. Population pairs using the same host (left, e.g., two populations in different geographic location that both use *Ceanothus*) are not exposed to divergent selection and show no progress towards speciation. Ecotype pairs (center) are exposed to divergent selection along a single axis (crypsis), and show only partial progress towards speciation. Species pairs (right) are exposed to divergent selection along both axes.

We found that the species pair examined here was more phenotypically and evolutionarily divergent than previously studied ecotype pairs, and that the species pair was also subject to divergent selection on a greater number of niche dimensions. The findings suggest that selection on a greater number of niche dimensions promotes evolutionary divergence. Of course, replication of the data reported here is required before the robustness and generality of our findings can be known. This is especially the case because only a single species pair was examined. Nonetheless, the level of replication reported here is typical of studies of ecological speciation (due in part to difficulties in implementing the necessary field experiments) [15], [16], [18], and the collective findings suggest a tentative and testable model for the diversification of *Timema* stick-insects (Fig. 3). The model is as follows. Pairs of populations using the same host-plant species exhibit little or no reproductive isolation, and differ along neither the niche dimension of crypsis nor that of physiology. Phenotypic divergence and speciation can be initiated by shifts in host-plant use, which first results in divergent selection on crypsis. However, divergent selection on the single dimension of crypsis may be insufficient to complete speciation. Greater adaptive divergence and reproductive isolation might be observed only when selection on crypsis is coupled with selection on the additional dimension of physiology. Future work in *Timema* should focus on why host plant shifts sometimes result in selection on multiple dimensions, but other times do not. Such work would be particularly informative given the taxon pairs studied here have diverged in the same host-plant genera, yet differ in the number of niche dimensions subject to divergent selection.

Our findings also provide some preliminary information on the temporal order of evolution of different traits during evolutionary divergence. Specifically, the results suggest that colouration differences may evolve first, followed by physiology (although again, further data from additional taxon pairs is required to substantiate this hypothesis). If visual predation is intense following the colonization of a new host species, a very low proportion of individuals may survive long enough to be subject to selection on physiological traits. This raises the interesting possibility that physiological adaptation is more likely when predation is weak. The results also suggest that some host preference evolution can occur via selection on crypsis alone, before (i.e., without) physiological adaptation, because the ecotypes do exhibit weakly divergent host preferences despite being subject only to divergent selection on crypsis. Nonetheless, stronger preference divergence might require selection on both crypsis and physiology, as observed in the species pair examined here.

In some sense, it is not surprising that the species pair was more phenotypically and evolutionarily divergent than the ecotypes within species, and was also subject to selection on more niche dimensions. However, we stress that this need necessarily be the case. For example, the increased phenotypic divergence and progress towards speciation could have had nothing to do with selection on an additional niche dimension (in this case physiology), but instead could have been related to any number of other factors, including stronger selection on a single dimension (in this case crypsis) [17]–[19], [21], [71], non-host plant related selection [15], [80], the opportunity for genetic drift [17], [21], the geographic arrangement of populations [21], or the genetic basis of the traits under selection [18], [47]–[55]. This is particularly the case because distinct species pairs of herbivorous insects that use different host plant species but do not exhibit physiological trade-offs between hosts are known [73], [74].

A number of other factors warrant consideration when interpreting our results. First, there are interesting issues related to the interface of polymorphism maintenance and speciation. We argued above that our conclusions do not depend on whether the ecotypes are differentiating further, or if they represent a ‘polymorphism’ that is maintained within species for a long period of time (the age of the ecotype pairs supports the latter interpretation, see below). Nonetheless, it is of interest to consider the maintenance of polymorphism, and the potential contribution of frequency-dependent selection to this process. Frequency dependent selection has been shown to be important for the maintenance of morphs in damselflies [38], [39], lizards [35], guppies [82], [83], and other organisms [reviewed by 37], [39]. Although there is no direct data in *Timema*, some role for frequency dependence is suggested by the observation that maladaptive (i.e., less cryptic) morphs are maintained at low frequencies within allopatric populations for long periods of time [68], [69], [70], [77], [84]. This could occur via a number of mechanisms (e.g., occasional gene flow into allopatry) [84], but one pertaining to frequency dependence is increased shelter from predation for rare, less cryptic morphs, via the formation of a search image by predators for more common (but more cryptic) prey [85]. However, a role for frequency dependent selection that outweighs that of divergent selection for explaining patterns of divergence is unlikely given that: 1) morphological traits (colour, size, shape) are strongly related to host plant use (i.e., divergent selection), 2) colour morph frequencies are stable through time, at the scale of months, years, and even decades, with no evidence for strong temporal oscillations [68], [69], [70], [84], 3) colour is often monomorphic within host species, precluding frequency dependent selection within hosts (e.g., *T. chumash* is always green, *T. podura* on *Adenostoma* is always brown), and 4) when polymorphism within hosts does occur, a process other than frequency dependence, namely gene flow between hosts, is known to play a central role in generating and maintaining variation [68], [69], [70], [77], [84]. However, we certainly do not rule out some role for frequency dependence, and further studies focused on it would be of interest.

Another issue pertaining to polymorphism maintenance is the evolution of genetic dominance. Selection might favor the evolution of dominance among alleles, making heterozygotes more similar to one of the homozygotes [36]–[39]. Resembling a homozygote has advantages under both frequency-dependent disruptive selection within populations and under divergent selection between environments. In the former, homozygotes have higher fitness because they are rare [36], [39]. In the latter, one homozygote has the highest fitness in each environment (i.e., intermediates do poorly in both environments, and each homozygote is best adapted to its native versus the alternative environment) [18]. Thus, there may be a race between how fast dominance versus reproductive isolation between sympatric morphs evolves [36], [39]. A good understanding of the extent to which such processes occur in *Timema* awaits more detailed data on the genetic basis of the traits under selection. Some preliminary insight does exist. One of the colour-pattern elements in *T. cristinae* (the presence versus absence of a dorsal stripe) does appear to be controlled by a single Mendelian locus with dominance of the unstriped allele [70], [79]. However, dominance is incomplete and other traits in this species, such as host preference, appear to have a more additive, polygenic basis [78]. The genetic basis of host adaptation in *T. podura* and *T. chumash* is unknown (and neither species exhibits the stripe that *T. cristinae* does). We do note that there is little or no evidence for sexual dimorphism of colour traits in *Timema* [68], [69], [70]–[72], [79], suggesting that sex-limited expression is not involved in polymorphism maintenance. Further genetic data will help elucidate the extent to which selection and polymorphism maintenance affect the evolution of genetic architecture.

We note that the species pair examined here are not sister species. We thus focused on evolutionary divergence most generally, rather than the origination of the particular species pair examined. We considered different stages of evolutionary divergence, with post-speciational diversification being particularly relevant to divergence between non-sister species pairs such as the pair examined here. Nonetheless, we note that much has been learned about speciation by studying taxon pairs that are not sister taxa. For example, consider the seminal paper by Coyne and Orr [86] that plotted levels of reproductive isolation between species pairs of *Drosophila* against genetic distance (a proxy for time since divergence). Most of the species pairs examined were not sister species, yet this study generated influential insight into the evolution of reproductive isolation during the process of speciation. The study confirmed empirically the hypothesis that reproductive isolation increases with time, and also showed that premating isolation tends to be accentuated in sympatry versus allopatry (thereby rekindling enthusiasm for the controversial theory of reinforcement speciation) [17]. A suite of similar articles in disparate taxa (also using non-sister species pair) emerged since the original *Drosophila* work [reviewed in 17]. A recent study added data on ecological divergence to all these previously published studies of the association between reproductive isolation and genetic distance [19]. That study found a consistent positive association between reproductive isolation and ecological divergence, independent from time, across the disparate taxa studied to date. The results suggest that ecological divergence is a taxonomically general promoter of speciation. In short, much has been learned about speciation using non-sister taxa, by analyzing the causes of reproductive and evolutionary divergence.

Thus, our current work does provide some insight into speciation specifically, especially when the history of host plant use in the genus *Timema* is considered. Ancestor state reconstructions on a mitochondrial DNA phylogeny indicate that the most likely ancestral condition in the genus *Timema* was the use of both *Ceanothus* and *Adenostoma* (e.g., the root of phylogeny was reconstructed as a generalist using both these host species) [67], [81 for details]. Thus, a plausible phylogenetic scenario for divergent host plant adaptation is that ecotypes of *T. cristinae* and ecotypes of *T. podura* have been adapting to these two different hosts for quite some time, whereas *T. chumash* lost the use of *Adenostoma* and became specialized to *Ceanothus* (potentially resulting in the additional selection on physiology reported here, and contributing to the divergence of *T. chumash* from it's close relatives).

A final interesting question concerns the role of time since divergence. Some observations suggest that time does not play a large role in explaining the collective results in *Timema*. For example, the ecotypes of *T. cristinae* have not completed speciation, yet molecular data indicates that they are relatively old (or at least not extremely recent). For example, allopatric population pairs of the *T. cristinae* ecotypes exhibit mitochondrial (4% at COI) and nuclear (2% at ITS-2) DNA sequence divergence consistent with up to two millions years since the initiation of population divergence, and they also exhibit substantial F_ST_ values at AFLP loci (mean F_ST_ = 0.09) [75], [76], [80], [81]. Moreover, levels of reproductive isolation between populations of this species are uncorrelated with neutral genetic divergence (a proxy for time) [33], [75], [76]. A good estimate of the age of the species pair *T. podura* and *T. chumash* awaits further data [81]. Thus, although increased dimensionality of niche divergence may play a causal role in driving phenotypic divergence and progress towards speciation, the role of time in allowing such increased dimensionality deserves further study. Experiments with very recently formed species pairs, perhaps younger in age than the ecotypes, could address this issue. This raises some further points about inferring causality: increased dimensionality of niche divergence might promote speciation, reduced gene flow might allow divergence in a greater number of niche dimensions, or these two processes feed back on one another [77], [87], [88]. Two arguments indicate that the causal arrow lies, at least to some extent, in the direction of selection on more niche dimensions promoting speciation. First, we measured actual selection, rather than simply phenotypic divergence, and the former might be less affected by gene flow [84]. Second, allopatric population pairs using different hosts exist within all three taxon pairs. Such populations likely undergo little or no gene flow from the alternative host [33], [68], [70], [76]–[79], [84], indicating that gene flow is unlikely to constrain their niche divergence, and conversely, that niche divergence promotes speciation.

Although further studies are required to tease apart the role of time and causal associations, our results clearly show that for the few *Timema* taxa examined so far, the dimensionality of selection is positively associated with the degree of evolutionary divergence in nature. Comparable examples from other taxa are sparse, but recent evidence based upon levels of phenotypic divergence (rather than actual estimates of selection), or detailed consideration of selection on a singe niche dimension, does exist. For example, diapause life history traits among *Rhagoletis pomonella* group flies are likely under multifarious selection related to pre- and post-winter conditions, creating a stronger ecological barrier to gene flow [64]. In Lake Victoria cichlids, the degree of neutral genetic divergence between a sympatric species pair along a transect (a proxy for the degree of reproductive isolation) is related to the number of different types of phenotypic traits that have diverged between the species pairs (e.g., habitat choice behavior as inferred from water depth and distance from shore in the lake, diet inferred from stable isotopes, male aggression, parasites, and divergence in opsin genes affecting colour vision) [65], [89]–[91]. Importantly, these results from natural populations support those from experimental evolution studies, suggesting that dimensionality of selection may be a general complement to widely-considered genetic and geographic explanations for variability in the degree and rate of evolutionary diversification. Different stages of evolutionary divergence are evident in many taxa, and quantification of the dimensionality of niche divergence between them will allow tests of the generality of the niche dimensionality hypothesis.

## Materials and Methods

### Colour measurements

Body and stripe brightness were estimated from digital photographs, using previously published procedures [71]. Brightness contrast was calculated as body brightness minus stripe brightness. The data for *T. cristinae* stem from a previous study, whereas all the data for *T. chumash* (n = 164) and *T. podura* (n = 41, 9 for *Ceanothus* and *Adenostoma* respectively) were collected for the current study.

### Study Populations

The field experiments used individuals from a site in Southern California, which was about 200 m×200 m square. All the experiments used only individuals from this site. *Timema* were captured by sampling randomly throughout the entire site using sweep nets. The physiology experiment was conducted at Poppet Flat, whereas the perturbation experiment was conducted at a site several hundred meters away (and the intervening area had burned the previous year, such that movement between sites was unlikely, especially given the low dispersal ability of these wingless insects, estimated at 12m per generation on average) [92]. All statistical analyses (described hereafter) used two-tailed probabilities.

### Host-plant preferences

In March 2007, host-plant preferences of both species were assayed using procedures applied in past studies [72], [78], [79]. Individual walking-sticks were placed in the bottom of a 500 ml plastic cup (height, 15 cm), with one 12cm host cutting from each host-plant species in the cup (n = 33 *T. podura* and 29 *T. chumash*). The top of each container was covered with mesh, secured by elastic bands. These assays were initiated in the evening and test animals were left in darkness overnight (the insects feed nocturnally). In the morning, we recorded which host species each individual was resting on. Each individual was used only once and the branches of each host species were paired by collection site within each cup. A chi-squared test was used to determine whether the two species differed in their host plant preferences. In the field, *T. podura* was occasionally captured on *Ceanothus*, whereas extensive collecting by both authors never resulted in the capture of *T. chumash* on *Adenostoma*.

### Predation experiment

A manipulative perturbation experiment was conducted to test for divergent selection from visual predators. Procedures were similar to a past study [70]. The experiment was initiated in early March 2007. There were four treatments, *Ceanothus* versus *Adenostoma*, in the presence versus absence of visual predators, where avian predators were excluded using chicken-wire enclosures (3 cm mesh such that insects could disperse from both treatments). Using a total of 40 bushes (10 per treatment), we removed all the *Timema* from a bush, by shaking the bush vigorously until no *Timema* were captured in sweep nets after 15 minutes of shaking. Sample bushes were separated from all other suitable host plants by a minimum distance of 5m. Upon each individual bush, we then placed 10 individuals of each insect species. Four weeks later, we recorded the frequency of each species on each bush. This was done by placing a white sheet underneath the bush, visually inspecting the bush for *Timema*, and then shaking each branch such that any undetected insects would fall onto the sheet. A recapture session was considered complete when no walking-stick insects were found after 15 minutes of shaking the branches of a particular bush. ANOVA tested whether the final proportion of individuals that were *T. chumash* was dependent upon host species, presence versus absence of predators, or an interaction between these two factors.

### Physiology experiment

A reciprocal-transplant experiment was conducted to test for physiological trade-offs in host-plant use, using procedures similar to a past study [72]. We raised field-collected newborn nymphs inside of mesh enclosures (which exclude vertebrate predators), on each host in their natural habitat. A randomized block design was used, yielding four different treatments within each individual block, in a 2×2 factorial design (*T. podura* transplanted to both hosts, and *T. chumash* transplanted to both hosts). In each of 30 blocks, there was one shrub of each host species, separated by less than 2 m. Within each treatment for a given block, two newborn individuals of the same species were added to a fine mesh bag that enclosed a branch of the food plant sufficiently large (40 cm×60 cm) to support them until maturity. A cup of soil was added because *Timema* coat their eggs with soil. Every block was 3–10 m from its nearest neighbor with the farthest blocks approximately 200 m apart. The experiment was set up from March 4–17, 2007. We recorded the number of insects alive within each enclosure on April 22, May 7, May 21, June 3, and August 4. In the last census, all the insects had died, so the bags were collected and the eggs within them counted. Mean survival was estimated as the number of insects observed alive within an enclosure, averaged across the multiple census periods. Lifetime fecundity was the number of eggs within an enclosure after the final census. These two measures of fitness are dependent on one another, but the latter does not apply to males. Because our specimens were too young to sex when they were released into the enclosures, each enclosure may have included zero, one, or two females. However, this variation was completely random with respect to treatment, and thus cannot confound our results (and our survival data is less prone to this issue than the fecundity data).

We analyzed mean survival and lifetime fecundity using ANOVA (separate analyses were run for each measure of fitness). The models included two fixed factors with two levels each: (1) insect species, a ‘FROM’ factor with levels *T. podura* or *T. chumash* and (2) host transplanted to, a ‘TO’ factor with levels *Ceanothus* or *Adenostoma*. The models also included a random factor (block), and interaction terms. A significant FROM * TO interaction indicates that the effect of host species on fitness is dependent on the insect species, indicative of local adaptation and fitness trade-offs. We report the results from both a full-factorial model, and from best-fit models inferred using Akaike Information Criteria (AIC) coefficients [93]. In the latter approach, AIC coefficient are used to select the linear model that best fit the data, and the significance of the terms in the optimal model is tested using a general linear model [following 84].
